# Association between wait time of central venous pressure and 28-day mortality in critically patients with acute pancreatitis: A restrospective cohort study

**DOI:** 10.1097/MD.0000000000039438

**Published:** 2024-08-30

**Authors:** Ying Lan, Lvlin Chen, Qilin Yang, Bin Zhu, Zhimei Lin

**Affiliations:** a Department of Critical Care Medicine, Affiliated Hospital of Chengdu University, Chengdu, China; b Department of Critical Care, The Second Affiliated Hospital of Guangzhou Medical University, Guangzhou, China; c Hubei University of Science and Technology, Xianning, China; d Department of Hematology, Affiliated Hospital of Chengdu University, Chengdu, China.

**Keywords:** 28-day mortality, acute pancreatitis, central venous pressure, MIMIC-IV, wait time

## Abstract

Hemodynamic management is crucial in patients with acute pancreatitis. Central venous pressure (CVP) is widely used to assess volume status. Our aim was to determine the optimal time window for obtaining CVP measurements to prevent adverse outcomes in patients. This study utilized data from the Medical Information Mart for Intensive Care (MIMIC) IV database. The primary outcome under investigation was the 28-day mortality, while secondary outcomes included 90-day mortality and 1-year mortality. To categorize the study population, a CVP waiting time of 12 hours was employed as the grouping criterion, followed by the utilization of Cox regression analysis to compare the outcomes between the 2 groups. Our study included a total of 233 patients, among whom 154 cases (66.1%) underwent CVP measurements within 12 hours after admission to the Intensive Care Unit (ICU). Univariate and multivariate Cox regression analyses revealed a significantly increased risk of 28-day mortality in patients from the delayed CVP monitoring group compared to those who underwent early CVP measurements (HR = 2.87; 95% CI: 1.35–6.13; *P* = .006). Additionally, consistent results were observed for the risks of 90-day mortality (HR = 1.91; 95% CI: 1.09–3.35; *P* = .023) and 1-year mortality (HR = 1.84; 95% CI: 1.09–3.10; *P* = .023). In the ICU, an extended waiting time for CVP measurements in patients with acute pancreatitis was associated with an increased risk of 28-day mortality.

## 1. Introduction

Acute pancreatitis (AP) stands as a prevalent digestive ailment with the potential for severe morbidity and mortality.^[1]^ Despite its increasing global incidence, the underlying reasons remain unclear.^[2]^ This condition can induce a substantial release of proinflammatory cytokines from the pancreas, precipitating a systemic inflammatory response syndrome and multi-organ failure.^[3]^ Approximately 15% to 20% of AP cases are severe or necrotic, leading to mortality rates ranging from 20% to 40%.^[4]^ In the case of AP patients, early fluid resuscitation has been linked to enhanced clinical outcomes.^[5–7]^ Nonetheless, the risk of fluid overload looms, with the potential to exacerbate conditions such as pulmonary edema, respiratory failure, acute kidney injury, and elevated intraabdominal pressure.^[8–11]^

Central venous pressure (CVP) has consistently served as the primary indicator guiding fluid resuscitation in critically ill patients.^[12–14]^ In recent years, the utility of CVP has been brought into question due to its susceptibility to influences from chest, pericardial, and abdominal pressures.^[15]^ Nevertheless, both “extreme” CVP values and the dynamics of CVP measurements prove valuable in predicting fluid responsiveness.^[15,16]^ Recent investigations^[17–19]^ have demonstrated that early CVP measurements correlate with improved patient outcomes. However, the overall impact of CVP monitoring on patients with AP remains uncertain. Consequently, we undertook a retrospective cohort study to elucidate the relationship between the initiation delay of CVP measurements and 28-day mortality in AP patients.

## 2. Materials and methods

### 2.1. Study design

The cohort study was conducted using the critical care database in the Medical Information Mart for Intensive Care (MIMIC)-IV. For a detailed account of MIMIC-IV, please refer to a distinct source (15). MIMIC-IV comprises 76,540 Intensive Care Unit (ICU) admissions spanning from 2008 to 2019. One of the authors (BZ) obtained the necessary authorization (certification number 50374474) to access and oversee data extraction from the database. These data were de-identified beforehand, and the Institutional Review Boards of the Massachusetts Institute of Technology (No. 0403000206) and Beth Israel Deaconess Medical Center (2001-P-001699/14) approved the use of this database for research purposes. Additionally, we adhered to all relevant ethical regulations for the use of research data.

### 2.2. Selection of patients

Patients within the MIMIC-IV database who met the criteria for AP were considered eligible for inclusion. The definition of AP adhered to the Atlanta criteria, as previously described in the literature. Exclusions were made for patients falling into the following categories: those aged below 18 years, cases where CVP monitoring was not conducted. Additionally, our analysis was restricted to the first hospital admission and the initial ICU stay. Data related to patients with subsequent ICU admissions were excluded. Patients who completed their initial CVP measurements within 12 hours after ICU admission were classified into the early CVP group, while the remaining patients formed the delayed CVP group.

### 2.3. Variable extraction

The primary exposure in this study was the waiting time for CVP measurement, defined as the total duration in hours from ICU admission until the initial CVP measurement. Only the first CVP measurement for each patient was considered in the analysis. Data on age, sex, year of admission, sequential organ failure assessment (SOFA) score and simplified acute physiology score (SAPS II score) were gathered during the first 24 hours following admission to the ICU. Other essential indicators, such as heart rate, breathing rate, and average arterial pressure, were also obtained. Within the initial 24 hours of ICU admission, various laboratory examinations were conducted, including assessments of white blood cell (WBC) count, hemoglobin levels, platelet counts, glucose creatinine, lactate, sodium, and potassium. If a variable was recorded multiple times during this period, the most severe value was used. Furthermore, other health conditions such as heart failure, chronic respiratory disease, diabetes and renal disease were recorded.

### 2.4. Outcomes

The primary outcome of this study was the 28-day mortality. Secondary outcomes included the 90-day mortality and the 1-year mortality.

### 2.5. Statistical Analysis

Descriptive analyses were conducted for all participants. Continuous variables with a normal distribution were presented as means (standard deviations), while non-normally distributed continuous variables were expressed as medians (interquartile ranges [IQRs]). Categorical variables were represented as frequencies with corresponding percentages. To compare categorical variables, we employed the Chi-square test, while for normally distributed continuous variables, the *t*-test was utilized, and for nonnormally distributed continuous variables, the Kruskal–Wallis test was applied. To ascertain survival curves, we utilized Kaplan–Meier analysis along with the log-rank test.

We employed multivariate regression to delineate the association between CVP measurements and 28-day mortality. In accordance with clinical expertise and previous literature, we incorporated the following covariates into the multivariate regression model: age, gender, SOFA score, use of mechanical ventilation, use of vasopressors, comorbidities, vital signs (MAP, heart rate, oxygen saturation), biochemical parameters (hematocrit, potassium, sodium, creatinine, BUN), and initial lactate levels. To avoid bias caused by missing data, the primary outcome analysis was conducted after replacing missing values.

All analyses were performed using the statistical software packages R 4.3.1 (http://www.R-project.org, The R Foundation) and Free Statistics software versions 1.9.^[20]^ Two-tailed tests were applied, and a significance level of *P* < .05 was considered statistically significant.

## 3. Results

### 3.1. Baseline characteristics

**Figure 1.**
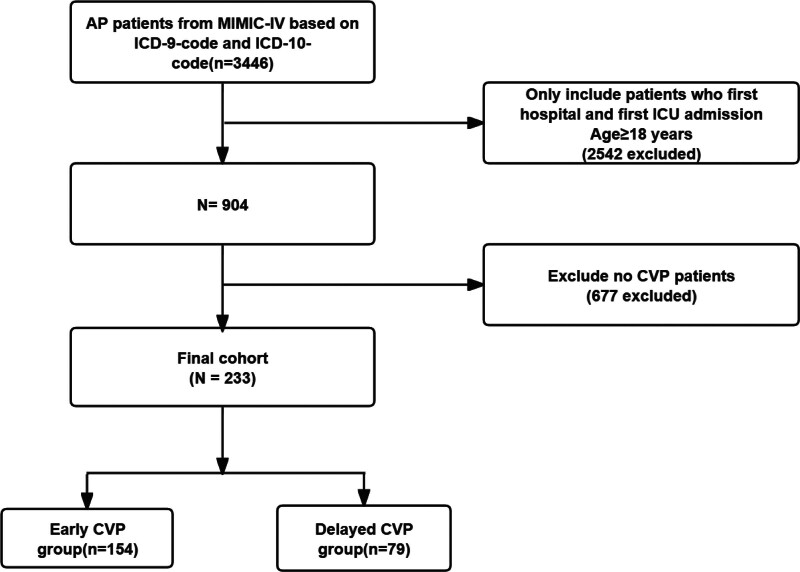
Study flow diagram in the present study.

After a thorough examination of the records from the MIMIC-IV database, which included 76,540 ICU patients, we identified and included a total of 233 patients for the current study. The selection process is outlined in Figure 1. In the study cohort, 154 patients (66.1%) underwent CVP measurements within 12 hours of ICU admission, with a median value of 12 mm Hg (IQR, 8–16 mm Hg); the time to the first CVP measurement was 3 hours (IQR, 1.7–5.7 hours). The baseline characteristics of all subjects are presented in Table 1. The participants had a mean age of 61.8 ± 16.4 years, with 131 (56.2%) being male. The early CVP group had a higher rate of mechanical ventilation use compared to the delayed CVP group (62.3% vs 47.2%, *P* = .034). However, there was no statistically significant difference in the use of vasopressor medications between the 2 groups. Overall, the baseline characteristics between the 2 groups were generally balanced.

**Table 1:** Baseline characteristics of participants.

Variables	All patients (n = 233)	CVP wait time		*P*
		Early (<12 h) (n = 154)	Delayed (≥12 h) (n = 79)	
Age (yr), mean (SD)	61.8 ± 16.4	61.6 ± 15.8	62.1 ± 17.6	.84
Male, sex, n (%)	131 (56.2)	87 (56.5)	44 (55.7)	.908
Vital signs, mean (SD)				
Heart rate (bpm)	118.6 ± 24.2	115.6 ± 22.4	124.4 ± 26.5	.008
MAP (mm Hg)	54.8 ± 17.6	53.6 ± 19.3	57.0 ± 13.6	.164
SpO_2_ (%)	90.1 ± 7.9	90.3 ± 8.7	89.6 ± 6.2	.534
Laboratory tests, median (IQR)				
WBC (10^9^/L)	15.7 (11.0, 22.1)	15.3 (10.0, 22.0)	16.5 (12.6, 22.2)	.141
Hematocrit (%)	35.0 (31.4, 40.3)	34.5 (31.0, 39.8)	36.2 (32.2, 40.8)	.174
Hemoglobin (g/L)	9.9 (8.6, 11.6)	9.9 (8.7, 11.4)	10.0 (8.6, 12.1)	.314
Platelet (10^9^/L)	148.0 (97.0, 209.0)	139.5 (97.0, 194.5)	165.0 (103.0, 215.0)	.22
Glucose (mg/dL)	136.2 (111.6, 177.0)	135.1 (114.1, 177.4)	141.0 (106.1, 173.8)	.901
Creatinine (mg/dL)	1.7 (0.9, 3.2)	1.8 (1.0, 3.2)	1.7 (0.9, 3.0)	.395
BUN (mg/dL)	31.0 (18.0, 50.0)	31.0 (19.0, 50.8)	30.0 (17.5, 47.0)	.353
Sodium, mmol/L	140.0 (138.0, 143.0)	140.0 (137.0, 143.0)	140.0 (138.0, 143.0)	.309
Potassium, mmol/L	3.8 (3.4, 4.2)	3.8 (3.4, 4.2)	3.8 (3.5, 4.2)	.984
Lactate, mmol/L	3.0 (1.7, 4.1)	3.0 (1.9, 4.2)	3.4 (1.6, 3.8)	.491
Comorbidity disease, n (%)				
Congestive heart failure	45 (20.6)	26 (17.8)	19 (26.4)	.141
Cerebrovascular disease	17 (7.8)	11 (7.5)	6 (8.3)	.836
Chronic pulmonary disease	55 (25.2)	34 (23.3)	21 (29.2)	.347
Diabetes	8 (3.7)	4 (2.7)	4 (5.6)	.444
Renal disease	44 (20.2)	28 (19.2)	16 (22.2)	.598
Sepsis	194 (89.0)	127 (87.0)	67 (93.1)	.178
SAPS II score, median (IQR)	46.0 (35.0, 58.0)	46.0 (35.0, 58.0)	46.0 (33.0, 54.5)	.567
SOFA score, median (IQR)	8.4 (6.0, 11.0)	8.4 (6.0, 11.0)	8.4 (6.0, 10.0)	.346
First CVP level (mm Hg), median (IQR)	12.0 (8.0, 16.0)	12.0 (8.0, 16.0)	11.0 (8.5, 16.0)	.721
CVP wait time (h)	5.8 (2.3, 17.5)	3.1 (1.7, 5.7)	27.3 (17.4, 62.4)	<.001
Interventions, n (%)				
MV use (first 24 h)	125 (57.3)	91 (62.3)	34 (47.2)	.034
Vasopressor use (first 24 h)	159 (72.9)	112 (76.7)	47 (65.3)	.074
RRT use (first 24 h)	21 (32.8)	15 (37.5)	6 (25)	.303

Abbreviation: MAP, mean arterial pressure; MV, mechanical ventilation; RRT, renal replacement therapy; SAPSII, simplified acute physiological score II; SOFA, Sequential Organ Failure Assessment; WBC, white blood cell.

### 3.2. Primary outcome

**Figure 2.**
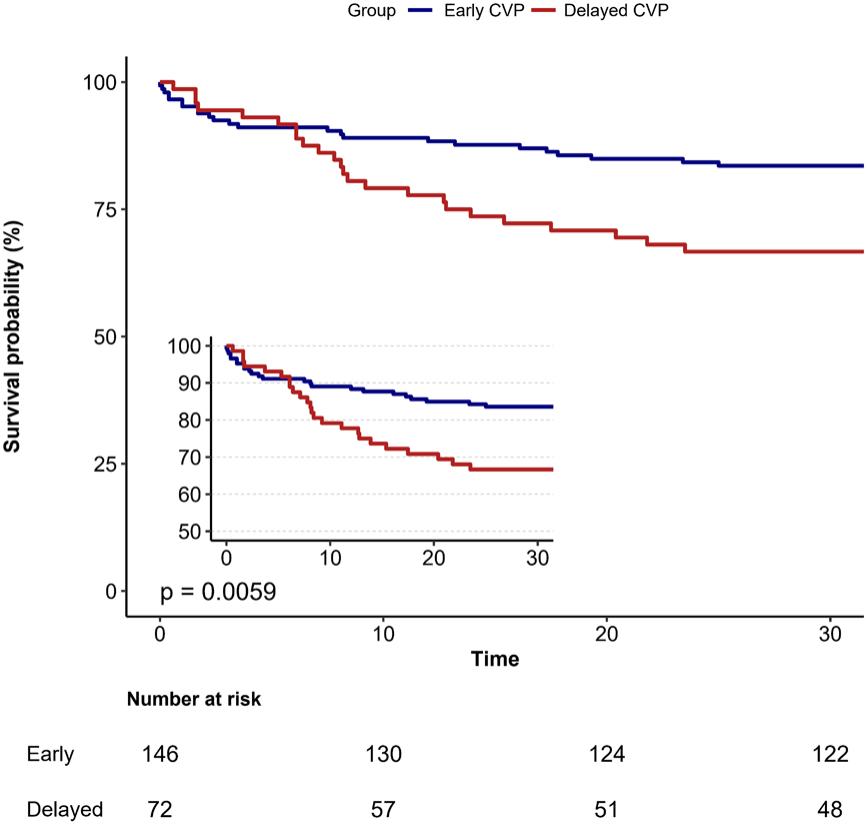
Kaplan–Meier survival analysis curves for 28-day mortality. Early (CVP wait time < 12 h), delayed (CVP wait time  ≥ 12 h).

Within the study, the 28-day mortality in the early CVP group and the delayed CVP group stood at 16.4% and 33.3%, respectively (*P* = .005) (refer to Table S1, Supplemental Digital Content, http://links.lww.com/MD/N425). According to the Kaplan–Meier curve, the delayed CVP group exhibited an increased 28-day mortality (log-rank test *P* = .0059, as shown in Figure 2). After adjusting for the full model, Cox regression analysis revealed that delayed CVP measurements were associated with an increased 28-day mortality, with a hazard ratio (HR) of 2.87 (95% CI: 1.35–6.13; *P* = .006) (Table 2).

**Table 2:** Associating CVP wait time with 28-day mortality in AP.

CVP wait time	Death (%)	Model 1		Model 2		Model 3	
		HR (95%CI)	*P* value	HR (95%CI)	*P* value	HR (95%CI)	*P* value
Early (<12 h)	24 (16.4%)	Ref		Ref		Ref	
Delayed (≥12 h)	24 (33.3%)	2.17 (1.23–3.83)	.007	2.25 (1.26–4)	0.006	2.87 (1.35–6.13)	.006

Note: Model 1: Unadjusted model; Model 2: Adjusted for age, gender, cerebrovascular disease, chronic pulmonary disease, diabetes, renal disease, congestive heart failure; Model 3: Model 2 plus heart rate, mean arterial pressure, glucose, WBC, HCT, SPO2, platelets, creatinine, BUN, SOFA score, lactate, potassium, sodium, MV use (first 24 h), Vasopressor use (first 24 h).

### 3.3. Secondary outcomes

The study demonstrated that, compared to the delayed CVP group, the early CVP group had a lower 90-day mortality rate (24% vs 38.9%, *P* = .022) and a lower 1-year mortality rate (28.1% vs 43.1%, *P* = .027), with statistically significant differences (Table S2, Supplemental Digital Content, http://links.lww.com/MD/N427). Similarly, consistent results were observed in terms of long-term mortality after adjusting for the full model. Cox regression analysis revealed that, compared to the early CVP group, delayed CVP measurements were associated with an increased 90-day mortality (adjusted HR = 1.91; 95% CI: 1.09–3.35) and 1-year mortality (adjusted HR = 1.84; 95% CI: 1.09–3.10) (refer to Table S2, Supplemental Digital Content, http://links.lww.com/MD/N427). Additionally, we observed a reduced incidence of AKI within 7 days among patients in the early CVP group (80.1% vs 93.1%, *P* = .013) (Table S1, Supplemental Digital Content, http://links.lww.com/MD/N425).

## 4. Discussion

Our study demonstrated a significant association between an extended CVP waiting time exceeding 12 hours following ICU admission and an elevated risk of 28-day mortality in patients with AP. Furthermore, consistent results were observed in terms of the risk of 90-day and 1-year mortality.

Yang et al^[19]^ found that among ICU patients with AKI, the early group (CVP measurements within 9 hours) exhibited lower adjusted in-hospital mortality compared to the delayed measurement group. Our study found that in patients with AP, early CVP measurements (within 12 hours) are associated with a reduced risk of 28-day and long-term mortality. Notably, the early CVP monitoring group exhibited a higher requirement for life support, including the use of mechanical ventilation and vasopressors. To mitigate potential confounding effects, we employed multivariate regression analysis, and the results remained consistent. However, the exact reasons for the link between early CVP measurement and reduced mortality in AP patients remain unclear.

ICU-admitted patients with AP frequently manifest adverse prognostic indicators, notably organ failure, accompanied by a marked elevation in mortality, as delineated in the study.^[21]^ The correlation between fluid resuscitation and the clinical course of pancreatitis has been established.^[22,23]^ Early and aggressive intravenous fluid resuscitation within 12 to 24 hours after onset was deemed most beneficial.^[24]^ Early goal-directed therapy (EGDT) was the most commonly used approach.^[25]^ CVP has long been widely employed to guide EGDT. Despite prior studies yielding contradictory evidence regarding the impact of CVP on critical outcomes, a better understanding of how CVP measurements influence clinical decision-making has emerged.^[17]^ In the treatment of AP, the concurrent presence of intraabdominal hypertension (IAH) and insufficient volume further increases the complexity of clinical decision-making regarding CVP.^[26]^ This might result in clinicians showing a less assertive utilization of CVP in patients with AP. In our retrospective study, we noted that among individuals undergoing CVP placement, 89% of patients with AP presented with concurrent sepsis. This intriguing observation may suggest that clinicians lean towards determining the need for CVP placement based on the presence of sepsis.

Research indicated that CVP measurements within the first 24 hours of ICU admission were associated with a reduced risk-adjusted 28-day mortality in septic patients.^[17]^ Yang et al found that among ICU patients with AKI, the early group (CVP measurements within 9 hours) exhibited a lower adjusted in-hospital mortality compared to the delayed measurement group.^[19]^ The correlation between the timing of CVP measurements and prognosis in patients with AP remains unclear. Our study revealed an association between early CVP measurements (within 12 hours) and 28-day as well as long-term mortality in patients with AP in the ICU. Further research was needed to elucidate the interpretation of this result. AKI increased the prognostic risk in AP, particularly in patients with concomitant ICU admission and sepsis.^[27]^ Both conditions of fluid overload and insufficient volume were correlated with an elevated risk of AKI.^[28–30]^ In our study, we observed that the early CVP monitoring group experienced reduced fluid input on the second and third days compared to the delayed group. This could be 1 of the reasons for the lower incidence of AKI in the early CVP group. However, there are multiple factors influencing the occurrence of AKI, with fluid being just 1 of them. Additionally, further research was needed to determine whether the occurrence of AKI mediates the association between CVP measurements and prognosis.

### 4.1. Limitations

Several limitations warrant consideration in our study. Firstly, akin to all retrospective analyses, the potential for residual confounding factors exists. To mitigate the impact of factors that could introduce bias into the results, we diligently adjusted for plausible confounding variables. Secondly, the CVP grouping may introduce some bias. We categorized patients based on previous literature. Thirdly, our study did not thoroughly explore the causal relationship between CVP measurement and 28-day mortality, and as such, the need for further research to validate our findings was evident. Additionally, due to the limitations of the MIMIC database, we were unable to extract specific causes of death for the patients, which also restricted our ability to conduct further in-depth analysis. Lastly, it was important to note that our investigation exclusively examined the time to the first measurement of CVP and did not delve into CVP levels. Furthermore, we needed to exercise caution regarding the generalizability of our study findings, as it was a single-center study. Consequently, subsequent studies should consider exploring the potential role of CVP levels in disease progression.

## 5. Conclusions

In conclusion, an extended waiting time for CVP measurement was correlated with an elevated risk-adjusted 28-day mortality in critically ill patients with AP.

## Acknowledgments

We would like to thank the team of the Laboratory for Computational Physiology from the Massachusetts Institute of Technology (LCP-MIT) for keeping the MIMIC databases available.

## Author contributions

**Data curation:** Qilin Yang.

**Writing – original draft:** Ying Lan, Lvlin chen, Bin Zhu.

**Writing – review & editing:** Qilin Yang, Zhimei lin.

## Supplementary Material


