# Supplementary material for: Association between wait time of central venous pressure and 28-day mortality in critically patients with acute pancreatitis: A restrospective cohort study
Source: Medicine (Baltimore). 2024 Aug 30;103(35):e39438. doi: 10.1097/MD.0000000000039438 (PMC11365617; doi:10.1097/MD.0000000000039438)
Supplement: Supplementary file 1 [file medi-103-e39438-s001.docx]

**Table S1** Clinical outcomes analysis

| **Outcomes** | **CVP wait time** | | **P value** |
| --- | --- | --- | --- |
|  | **Early(＜12h)**  **(n=154)** | **Delayed(≥12h)**  **(n=79)** |  |
| **Primary outcome** |  |  |  |
| 28-day mortality, n (%) | 24 (16.4) | 24 (33.3) | 0.005 |
| **Secondary outcomes** |  |  |  |
| 90-day mortality, n (%) | 35 (24) | 28(38.9) | 0.022 |
| 1-year mortality, n (%) | 41 (28.1) | 31(43.1) | 0.027 |
| AKI within 7 days, n (%) | 117(80.1) | 67(93.1) | 0.013 |
| Input day 1 (mL), Median (IQR) | 6102.2 (3833.1, 8813.1) | 5973.4 (3814.4, 9000.5) | 0.751 |
| Input day 2 (mL), Median (IQR) | 3059.4 (1480.4, 4899.9) | 3369.5 (2380.0, 6016.2) | 0.056 |
| Input day 3 (mL), Median (IQR) | 2303.1 (1254.0, 3842.4) | 2771.7 (1338.3, 4740.1) | 0.259 |

Abbreviation: AKI, acute kidney injury.
