# Supplementary material for: Association between wait time of central venous pressure and 28-day mortality in critically patients with acute pancreatitis: A restrospective cohort study
Source: Medicine (Baltimore). 2024 Aug 30;103(35):e39438. doi: 10.1097/MD.0000000000039438 (PMC11365617; doi:10.1097/MD.0000000000039438)
Supplement: Supplementary file 2 [file medi-103-e39438-s002.docx]

**Table S2** Associating CVP wait time with 90-day mortality and 1-year mortality in AP

| **Outcomes** | **Model 1** | |  | **Model 2** | |  | **Model 3** | |
| --- | --- | --- | --- | --- | --- | --- | --- | --- |
|  | **HR (95%CI)** | **P value** |  | **HR (95%CI)** | **P value** |  | **HR (95%CI)** | **P value** |
| **90-day mortality** |  |  |  |  |  |  |  |  |
| **CVP wait time** |  |  |  |  |  |  |  |  |
| **Early(＜12h)** | Ref |  |  | Ref |  |  | Ref |  |
| **Delayed(≥12h)** | 2.17 (1.23~3.83) | 0.007 |  | 1.95 (1.18~3.23) | 0.01 |  | 1.91 (1.09~3.35) | 0.023 |
| **1-year mortality** |  |  |  |  |  |  |  |  |
| **CVP wait time** |  |  |  |  |  |  |  |  |
| **Early(＜12h)** | Ref |  |  | Ref |  |  | Ref |  |
| **Delayed(≥12h)** | 1.73 (1.09~2.76) | 0.021 |  | 1.91 (1.19~3.07) | 0.007 |  | 1.84 (1.09~3.1) | 0.023 |

Note: Model 1: Unadjusted model; Model 2: Adjusted for age, gender, cerebrovascular disease, chronic pulmonary disease, diabetes, renal disease, congestive heart failure; Model 3: Model 2 plus heart rate, mean arterial pressure, glucose, WBC, SPO2, platelets, creatinine, sofa score, lactate, potassium, sodium, MV use (1^st^ 24 h), Vasopressor use (1^st^ 24 h).
